# Turing mechanism underlying a branching model for lung morphogenesis

**DOI:** 10.1371/journal.pone.0174946

**Published:** 2017-04-04

**Authors:** Hui Xu, Mingzhu Sun, Xin Zhao

**Affiliations:** 1 Institute of Robotics and Automatic Information System, Nankai University, Tianjin, China; 2 Tianjin Key Laboratory of Intelligent Robotics, Nankai University, Tianjin, China; Shanxi University, CHINA

## Abstract

The mammalian lung develops through branching morphogenesis. Two primary forms of branching, which occur in order, in the lung have been identified: tip bifurcation and side branching. However, the mechanisms of lung branching morphogenesis remain to be explored. In our previous study, a biological mechanism was presented for lung branching pattern formation through a branching model. Here, we provide a mathematical mechanism underlying the branching patterns. By decoupling the branching model, we demonstrated the existence of Turing instability. We performed Turing instability analysis to reveal the mathematical mechanism of the branching patterns. Our simulation results show that the Turing patterns underlying the branching patterns are spot patterns that exhibit high local morphogen concentration. The high local morphogen concentration induces the growth of branching. Furthermore, we found that the sparse spot patterns underlie the tip bifurcation patterns, while the dense spot patterns underlies the side branching patterns. The dispersion relation analysis shows that the Turing wavelength affects the branching structure. As the wavelength decreases, the spot patterns change from sparse to dense, the rate of tip bifurcation decreases and side branching eventually occurs instead. In the process of transformation, there may exists hybrid branching that mixes tip bifurcation and side branching. Since experimental studies have reported that branching mode switching from side branching to tip bifurcation in the lung is under genetic control, our simulation results suggest that genes control the switch of the branching mode by regulating the Turing wavelength. Our results provide a novel insight into and understanding of the formation of branching patterns in the lung and other biological systems.

## Introduction

The Mammalian lung is a striking example of organs that develop through branching morphogenesis. During lung morphogenesis, two primary forms of branching, side branching and tip bifurcation, which occur in sequence, have been identified[1]. The switch of branching mode from side branching to tip bifurcation is postulated to be under genetic control[1, 2].

To investigate how genes work to generate these patterns, a mathematical model[3] derived from the Gierer-Meinhardt activator-inhibitor model[4] was used in our previous study[5]. We demonstrated a mechanism through which the interaction of biological morphogens creates branched structures in the lung. The cascades of branching forms that have been observed in the lung, including side branching and tip bifurcation, were successfully reproduced by the branching model. Although the biochemical mechanism—the interaction of morphogens—provides an elegant explanation of lung branching morphogenesis, the mathematical mechanism underlying the branching patterns needs to be further investigated. For example, the branching mode switch between side branching and tip bifurcation can be controlled by a key parameter related to consumption by cells in the simulation of the model; however, it is not easily explained by the interaction of morphogens. Mathematical studies focus on the dynamical behaviors of mathematical models [6–9]. However, there is lack of bridge between branching morphogenesis and mathematical mechanism. Based on the branching model, we investigate the mathematical mechanism underlying lung branching pattern formation in this paper.

In our previous study of the dynamics of side branching and tip bifurcation[10], we showed that Turing instability occurs in the branching patterns. Turing instability can induce spatial patterns in the models, such as spots, stripes, hole patterns, and more complicated patterns, which is applied to modeling biological patterning phenomena in fish skin, terrestrial vegetation, sea shells, and others[11–14]. To reveal the mathematical mechanisms underlying branching patterns, we conducted Turing instability analysis.

In this paper, we decoupled an activator-inhibitor model from the branching model and performed simulations of the two models to obtain Turing patterns and branching patterns. Our simulation results show that Turing instability occurs at the growing tip of the branching patterns. The Turing patterns underlying the branching patterns are spot patterns. The spot patterns are in the form of concentration peaks, leading to branching patterns, with a local activator concentration peak formed and moving ahead of the growing tips. This indicates that the local morphogen concentration peak plays a key role in the growth of branching. Furthermore, a sparse spot pattern underlies the tip bifurcation patterns, while a dense spot pattern underlies the side branching patterns. The dispersion relation analysis shows that the wavelength of the spot patterns acts on the branching structures. As the wavelength decreases, the spot patterns change from sparse to dense, the rate of tip bifurcation decreases and side branching eventually occurs instead. A sufficient wavelength is required for the occurrence of tip bifurcation, while an insufficient wavelength provides favorable conditions for side branching. Our results suggest that genes control the branching structures in the lung by regulating the Turing wavelength. Our results provide a fresh insight into and understanding of the formation of branching patterns in the lung and other biological branching systems.

## Methods

The branching model we used in this paper is defined by Eqs (1)–(4). The four variables in the model equations are: activator A, inhibitor H, substrate S, and cell differentiation state Y.

$$\frac{\partial A}{\partial t}=\frac{cA^{2}S}{H}-\mu A+\rho_{A}Y+D_{A}\nabla^{2}A$$ (1)

$$\frac{\partial H}{\partial t}=cA^{2}S-vH+\rho_{H}Y+D_{H}\nabla^{2}H$$ (2)

$$\frac{\partial S}{\partial t}=c_{0}-\gamma S-\varepsilon YS+D_{S}\nabla^{2}S$$ (3)

$$\frac{\partial Y}{\partial t}=dA-eY+\frac{Y^{2}}{1+fY^{2}}$$ (4)

In the branching model, the term $\frac{cA^{2}S}{H}$ in the first equation represents that activator A is up-regulated by itself in autocatalytic reaction kinetics at rate *c*, with the dependence on substrate S, and inhibited by inhibitor H. *cA*^2^*S* in the second equation represents the catalyzed effect of H by A. *ρ*_*A*_*Y* and *ρ*_*H*_*Y* represent A and H are secreted by differentiated cells Y at rates *ρ*_*A*_ and *ρ*_*H*_. *c*_0_ in the third equation represents S is produced at rate *c*_0_. −*εYS* represents S is consumed by differentiated cells Y at a rate *ε*. $\;dA+\frac{Y^{2}}{1+fY^{2}}$ represents a high A-concentration induces irreversible cell differentiation (the Y-concentration goes from low to high). −*μA*, −*vH*, −*γS*, and −*eY* represent A, H, S, and Y decay in a first-order reaction at rates *μ*, *v*, *γ*, and *e*. A, H, and S are assumed to be diffusible, with diffusion coefficients *D*_*A*_, *D*_*H*_, and *D*_*S*_, respectively.

**Fig 1:**
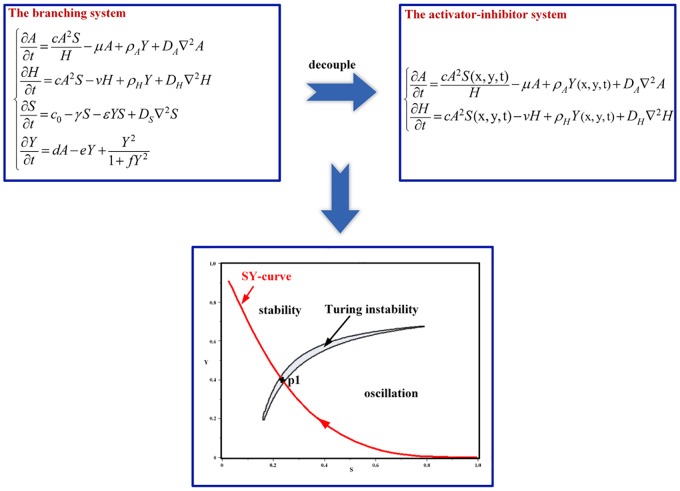
Schematic diagram of the research scheme. Top: The branching model was decoupled to obtain the activator-inhibitor model. Bottom: The S-Y parameter space of the activator-inhibitor model was calculated for the Turing instability. A crescent-shaped Turing region is presented as a result. The SY-curve for the differentiation trajectory of a cell in the branching system was plotted in the S-Y plane. Each state (S, Y) of cell differentiation is represented as a point on the SY-curve. To acquire the Turing pattern underlying the branching pattern, a point (e.g., point p1) on the trajectory within the Turing region was taken as the values of Sand Y, and simulation of the activator-inhibitor model was performed.

This branching model is derived by an activator-inhibitor system (Eqs 1 and 2) with dependence on the substrate. Thus, the branching pattern formation described in the model is divided into two processes: the formation of the local pattern on the stalk and the spatial extension of the stalk. The former is generated by the activator-inhibitor system, which exhibits Turing instability, and the latter results from the dependence of the activator-inhibitor system on the substrate. To explore the Turing instability underlying the branching model, a scheme was performed according to the following steps, as depicted in Fig 1.

Step 1. Decoupling. Decouple the activator-inhibitor model from the branching model, with S and Y as the parameters. We used the decoupling method described in the literature [10].
Step 2. Calculating a crescent-shaped Turing region. Calculate the S-Y parameter space of the activator-inhibitor model for the Turing instability (see Appendix for the Turing instability analysis).
Step 3. Plotting the differentiation trajectory of a cell. Extract (S, Y) pairs of a cell in the branching system with cell differentiation, then plot an SY-curve for the differentiation trajectory of the cell.
Step 4. Turing state selection and simulation. Select a point on the trajectory within the Turing region as the values of parameters S and Y and perform simulation of the activator-inhibitor model to obtain the Turing-type pattern underlying the branching pattern.

### Numerical simulation

We performed numerical simulations of both the branching model and the activator-inhibitor model to obtain the branching patterns and the related Turing patterns. For the branching model, we set the values of the parameters according to the literature [5]. For the activator-inhibitor model, simulations were performed on a 200×200 grid with periodic boundary conditions, and the parameter values and initial values of the variables were set according to the branching system. Starting from a randomly perturbed uniform initial condition, the simulation of the activator-inhibitor model was stopped when the stationary spatial structure was formed.

## Results

### Turing spot patterns underlying the branching patterns

**Fig 2:**
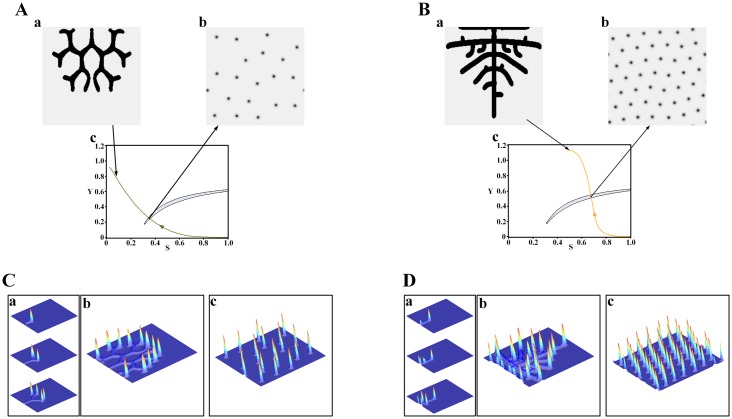
Turing spot patterns underlying the branching patterns. (A) For the tip bifurcation pattern (Aa), the underlying Turing pattern is a spot distribution (Ab), which appears at the point of the cell differentiation trajectory (Ac, green curve) within the crescent-shaped Turing region (Ac, gray shadow region). (B) Same as (A) but for the side branching pattern. The Turing pattern underlying the side branching pattern is also a spot distribution. (Ca) The growth of tip bifurcation in the tip bifurcation pattern (Aa) in 3D form. (Cb) The tip bifurcation pattern (Aa) in 3D form. (Cc) The underlying spot pattern (Ab) in 3D form. (Da) The growth of side branching in the side branching pattern (Ba) in 3D form. (Db) The side branching pattern (Ba) in 3D form. (Dc) The underlying spot pattern (Bb) in 3D form. Parameters: *c* = 0.002, *μ* = 0.16, *ρ*_*A*_ = 0.03, *D*_*A*_ = 0.02, *v* = 0.04, *ρ*_*H*_ = 0.0001, *D*_*H*_ = 0.3, *c*_0_ = 0.02, *γ* = 0.02, *D*_*s*_ = 0.06, *d* = 0.008, *e* = 0.1, *f* = 10, (Aa) *ε* = 1.0, (Ba) *ε* = 0.06, (Ab) *S* = 0.352, *Y* = 0.248, (Bb) *S* = 0.679, *Y* = 0.510. In the 2D patterns, black color indicates a high concentration, while gray indicates a low concentration. In the 3D patterns, red indicates a high concentration, while blue indicates a low concentration.

To explore the Turing patterns underlying the branching patterns, we calculated the S-Y parameter space for the Turing instability to obtain a crescent-shaped Turing region and recorded the cell differentiation trajectories of both the tip bifurcation and side branching patterns. We then performed simulations of their underlying Turing patterns. The simulation results are shown in Fig 2.

For the tip bifurcation patterns (Fig 2Aa), the underlying Turing patterns are spot distributions (Fig 2Ab). The spot patterns are at points on the cell differentiation trajectories of the tip bifurcation patterns within the Turing region (Fig 2Ac).The points refer to a cell state where cells are located at the growing tips of the tip bifurcation patterns. This means the Turing instability occurs at the growing tips of the tip bifurcation patterns.

To further investigate the effect of the spot patterns on the tip bifurcation patterns, we explored their activator distribution since the activator and inhibitor interaction plays a key role in pattern formation. In Fig 2C, the tip bifurcation patterns always exist with a local activator concentration peak that is formed and moves ahead of the growing tips (Fig 2Ca and 2Cb). The spot patterns are in the form of peaks of the activator concentration (Fig 2Cc). This indicates that the Turing instability affects the branching growth. The structure of the spot pattern leads to the activator concentration peaks formed at the growing tips of the tip bifurcation patterns.

**Fig 3:**
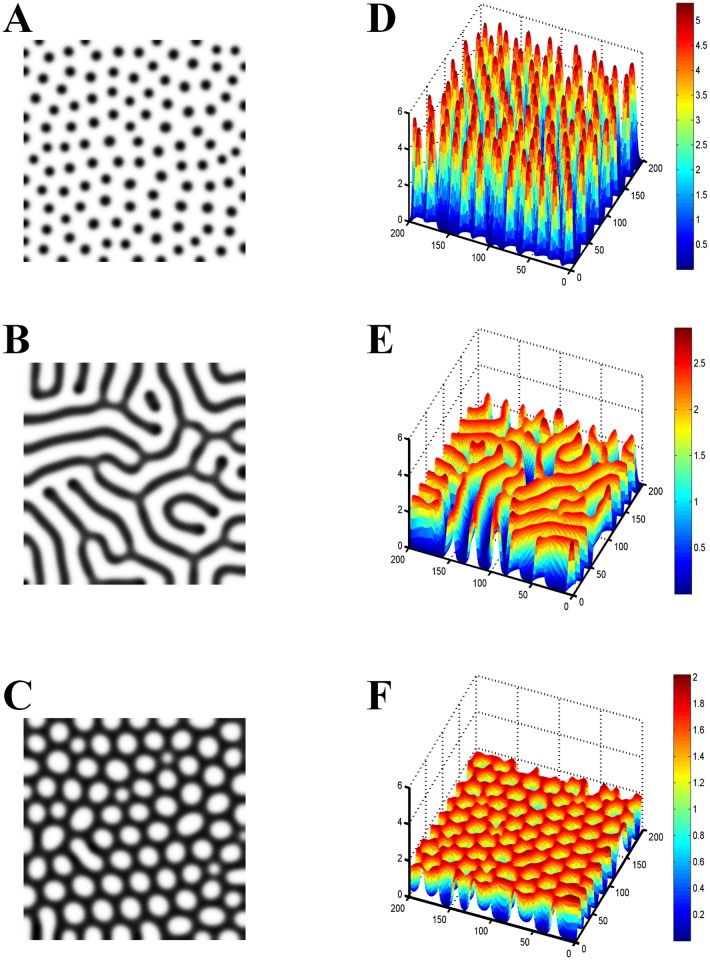
Comparison of the concentration gradients in different Turing patterns. (A) Spot pattern. (B) Stripe pattern. (C) Hole pattern. (D-F) The Turing patterns shown in 3D form corresponding to patterns A-C. The spot patterns exhibit the highest concentration gradient among the Turing patterns. The Turing patterns were generated by the activator-inhibitor model[4] with a saturation of activator production.

We then addressed how the Turing spot patterns underlie the tip bifurcation patterns. To interpret the mechanism, we explored the morphogen concentration in the Turing patterns because it plays an important role in cell growth[15, 16]. There are other typical structures of Turing patterns in addition to spots, such as stripes and holes. Fig 3 shows the activator concentration of those patterns. Spot patterns exhibit local high concentration peaks, while stripe and hole patterns are shown in a gentle concentration distribution. The spot patterns have a much higher activator concentration gradient than the stripe and hole patterns. This indicates that a high local morphogen concentration is required for tip bifurcation growth. The high concentration peaks at the growing tips of the tip bifurcation patterns caused by the Turing instability stimulate the outward extension of the tips and outward growth of the branches.

With respect to the side branching patterns (Fig 2Ba), the underlying Turing patterns also have spot distributions (Fig 2Bb). The spot patterns are at points on the cell differentiation trajectories of the side branching patterns within the Turing region (Fig 2Bc). The points refer to the cell state where cells are located at the growing tips of the side branching patterns, which means Turing instability also occurs at the growing tips for the side branching patterns. Fig 2D shows that a local activator concentration peak is formed and moves ahead of the growing tips of the side branching patterns (Fig 2Da and 2Db), which is consistent with the spot patterns in the form of peaks of the activator concentration (Fig 2Dc). Those results are the same as the case of the tip bifurcation patterns. However, the spot patterns underlying the side branching patterns are much denser than those corresponding to the tip bifurcation patterns.

### Spot density of the Turing spot pattern varies for branching structures

**Fig 4:**
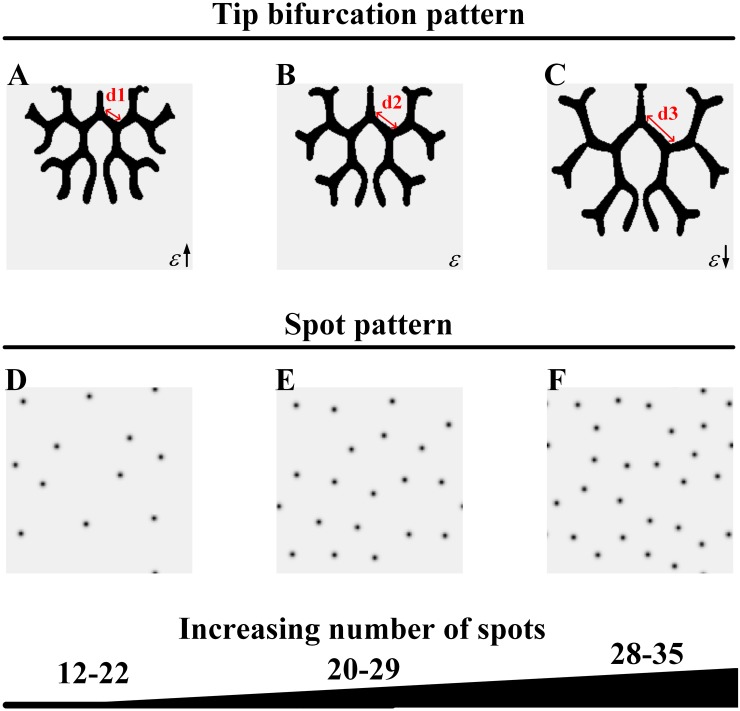
Sparse spot patterns underlying different tip bifurcation patterns. (A-C) The tip bifurcation patterns with increasing spatial interval between bifurcation events. (D-F) The underlying spot patterns have a sparse distribution, with increasing number of spots, which correspond to the tip bifurcation patterns A-C. Parameters: *c* = 0.002, *μ* = 0.16, *ρ*_*A*_ = 0.03, *D*_*A*_ = 0.02, *v* = 0.04, *ρ*_*H*_ = 0.0001, *D*_*H*_ = 0.3, *c*_0_ = 0.02, *γ* = 0.02, *D*_*S*_ = 0.06, *d* = 0.008, *e* = 0.1, *f* = 10, (A-C) *ε* = 1.5/1.0/0.7, (D-F) *S* = 0.320, *Y* = 0.185; *S* = 0.352, *Y* = 0.248; *S* = 0.395, *Y* = 0.313. In 2D patterns, black color indicates high concentration while gray indicates low.

**Fig 5:**
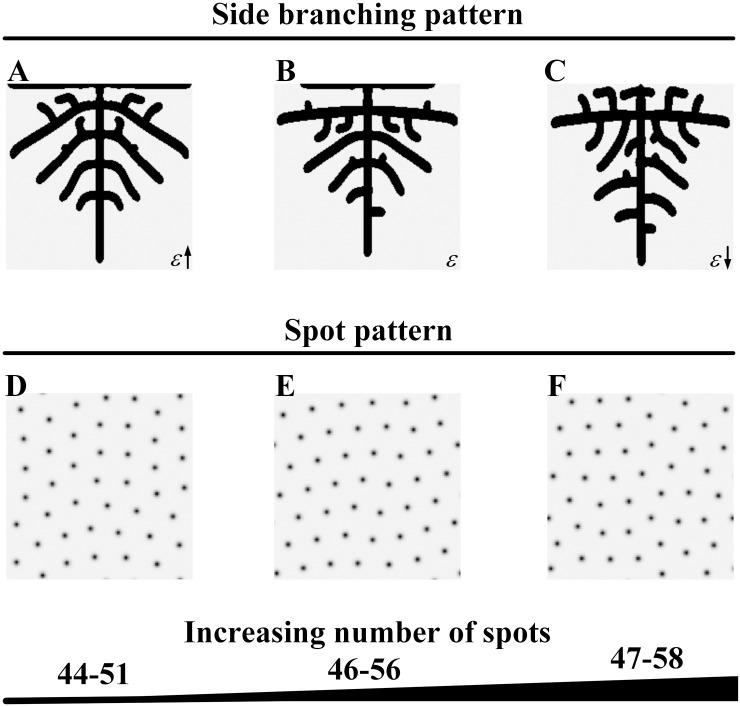
Dense spot patterns underlying different side branching patterns. (A-C) The side branching patterns. (D-F) The underlying spot patterns have a dense distribution that corresponds to the side branching patterns A-C. The spatial interval has a small decrease in the side branching patterns with the production of more branches as the number of spots slightly increases in the underlying spot patterns. Parameters: *c* = 0.002, *μ* = 0.16, *ρ*_*A*_ = 0.03, *D*_*A*_ = 0.02, *v* = 0.04, *ρ*_*H*_ = 0.0001, *D*_*H*_ = 0.3, *c*_0_ = 0.02, *γ* = 0.02, *D*_*S*_ = 0.06, *d* = 0.008, *e* = 0.1, *f* = 10, (A-C) *ε* = 0.1/0.06/0.045, (D-F) *S* = 0.614, *Y* = 0.478; *S* = 0.679, *Y* = 0.510; *S* = 0.716, *Y* = 0.530. In the 2D patterns, black indicates a high concentration while gray indicates a low concentration.

We observed that the structure of the Turing patterns underlying the branching patterns is spots. Furthermore, the spot density of the Turing patterns varies for the tip bifurcation and side branching patterns. Next, we investigated the connection between the spot density of the Turing spot patterns and the branching structures. In the simulation, both branching structures, tip bifurcation and side branching, can be generated by modifying a single parameter, *ε* (in Eq (3), the consumption rate of substrate by Y cells). In this way, we obtained the branching patterns and the underlying spot patterns, as shown in Figs 4 and 5.

#### Sparse Turing spot patterns underlying the tip bifurcation patterns

We set the tip bifurcation pattern shown in Fig 2Aa for a given *ε*. For convenience of comparison, we show the pattern in Fig 4B and its underlying spot pattern in Fig 4E. We then increased *ε* and obtained a tip bifurcation pattern with an increasing bifurcation rate, and the underlying spot pattern with a decreasing number of spots was observed (Fig 4A and 4D). Subsequently, we decreased *ε*, and another tip bifurcation pattern with a decreasing bifurcation rate were obtained, while the underlying spot pattern with an increasing number of spots was observed (Fig 4C and 4F).

For the tip bifurcation patterns, the underlying Turing patterns are sparse spot patterns. Tip bifurcation occurs at a decreasing rate with increasing number of spots in the spot patterns as *ε* decreases.

#### Dense Turing spot patterns underlying the side branching patterns

When *ε* is below a certain value, side branching patterns emerge rather than tip bifurcation patterns. We set the side branching pattern shown in Fig 2Ba for a given *ε*; we show the pattern in Fig 5B and its underlying spot pattern in Fig 5E. We then increased *ε* within the range for side branching patterns, and a side branching pattern was obtained with slightly increasing spatial interval between branches, while the underlying spot pattern was observed with a slightly decreasing number of spots (Fig 5A and 5D). Subsequently, we decreased *ε*, and another side branching pattern was obtained with a slightly decreasing spatial interval between branches and more outward growing branches, while the underlying spot pattern was observed with a the slightly increasing number of spots (Fig 5C and 5F).

For the side branching patterns, the underlying Turing patterns are dense spot patterns. As *ε* decreases, more side branches are produced, the spatial interval between branches decreases, and the number of spots in the underlying spot patterns increases.

### Turing wavelength regulates the branching structures

**Fig 6:**
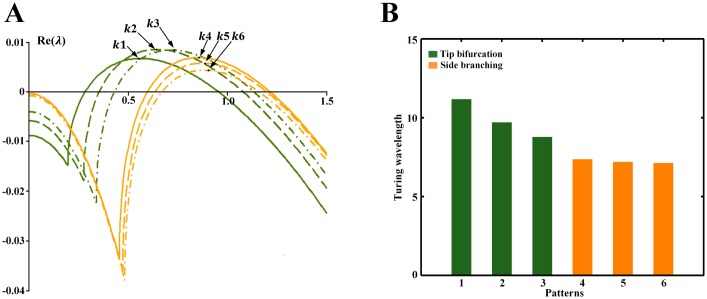
Dispersion relations for the spot patterns underlying the branching patterns in Figs 4 and 5. (A) Dispersion relations for the spot patterns underlying the branching patterns in Figs 4 and 5. *λ* represents the eigenvalue with the largest real part for a given wavenumber *k*. *k*1-6 are the critical wavenumbers at which the maximum value of *λ* occurs at. *k*1-3 are the wavenumbers of the spot patterns in Fig 4D–4F, while *k*4-6 are the wavenumbers of the spot patterns in Fig 5D–5F. (B) Comparison of the wavelength (2π/*k*) of the spot patterns underlying the branching patterns in Figs 4 and 5. Patterns 1–3 represent the spot patterns in Fig 4D–4F, while patterns 4–6 represent the spot patterns in Fig 5D–5F.

Turing patterns are characterized by a critical wavelength[17]. To elucidate the phenomenon of distinct spot densities of the spot patterns underlying the tip bifurcation patterns and side branching patterns, we further explored the critical wavelength of the spot patterns by dispersion relation analysis. The dispersion relations shown in Fig 6 describe a function of Re(*λ*) that depends on wavenumber *k*, where *λ* is the eigenvalue with the largest real part (see Appendix for obtaining the dispersion relations). The wavelength is calculated by dividing 2π by the critical wavenumber at which the maximum value of *λ* occurs.

In Fig 6, we show the dispersion relations for the spot patterns underlying the branching patterns depicted in Figs 4 and 5 and present a comparison of the wavelength (2π/wavenumber) in sequence. For the tip bifurcation patterns, when the bifurcation rate decreases and the number of spots in the underlying sparse spot patterns increases (Fig 4), the dispersion relations illustrate that the wavelength decreases (Fig 6A, green curves; Fig 6B, green bars). When the branching mode switches from tip bifurcation to side branching with the underlying spot pattern change from a sparse to dense distribution, the wavelength decreases further (Fig 6A, green to orange curves; Fig 6B, green to orange bars). For the side branching patterns, when the spatial interval between branches decreases with more outward growing branches, the number of spots in the underlying dense spot pattern increases slightly, and the wavelength decreases slowly (Fig 6A, orange curves; Fig 6B, orange bars).

These data suggest that as the wavelength decreases, the number of spots in the spot patterns increases, and tip bifurcation occurs at a decreasing rate. When the wavelength decreases below a certain value, side branching occurs rather than tip bifurcation.

**Fig 7:**
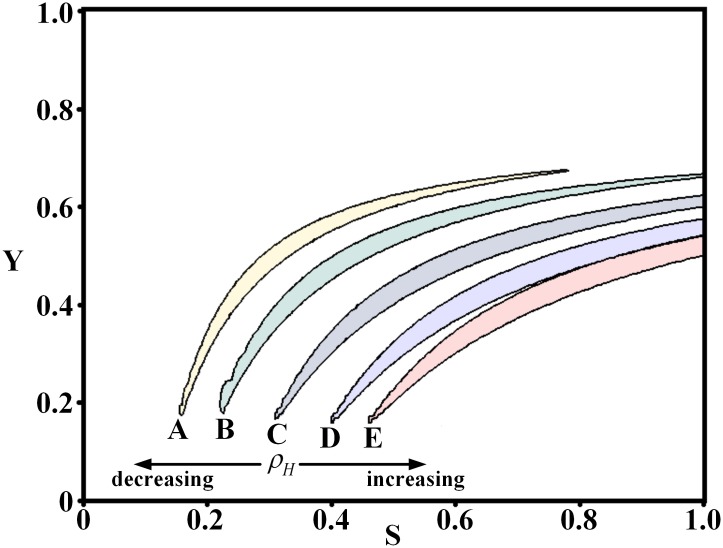
Five Turing regions obtained by varying *ρ*_*H*_. The Turing region C is the same region shown in Fig 2 for *ρ*_*H*_ = 0.0001. Turing regions A (*ρ*_*H*_ = 0.00005) and B (*ρ*_*H*_ = 0.00007) were obtained by decreasing *ρ*_*H*_. Turing regions D (*ρ*_*H*_ = 0.00013) and E (*ρ*_*H*_ = 0.00015) were obtained by decreasing *ρ*_*H*_.

**Fig 8:**
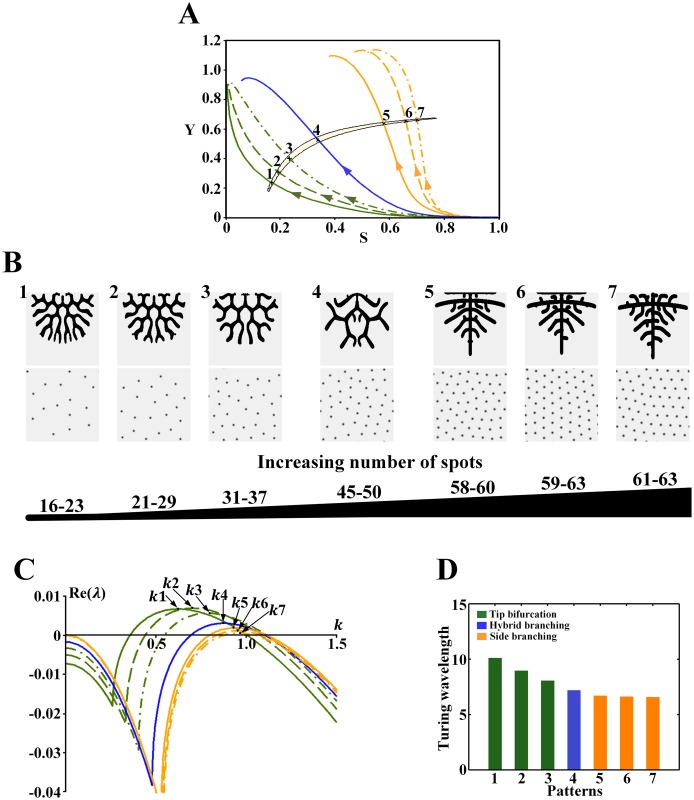
Effect of the wavelength on the branching patterns in the Turing region for *ρ*_*H*_ = 0.00005. (A) The cell differentiation trajectories of the branching patterns cross the Turing region. Points 1–7 correspond to the position of the underlying Turing patterns. (B) Branching patterns (top) and underlying spot patterns (bottom). Patterns 1–7 correspond to points 1–7 in A. (C) Dispersion relations for patterns, with wavenumber *k*1-7 corresponding to patterns 1–7 in B. (D) Comparison of the wavelength (2π/wavenumber) from patterns 1–7 in B. Parameters: *c* = 0.002, *μ* = 0.16, *ρ*_*A*_ = 0.03, *D*_*A*_ = 0.02, *v* = 0.04, *ρ*_*H*_ = 0.00005, *D*_*H*_ = 0.3, *c*_0_ = 0.02, *D*_*S*_ = 0.06, (B1-7) *ε* = 3.0/2.0/1.0/0.5/0.1/0.06/0.045. In the 2D patterns, black indicates a high concentration, and gray indicates a low concentration.

**Fig 9:**
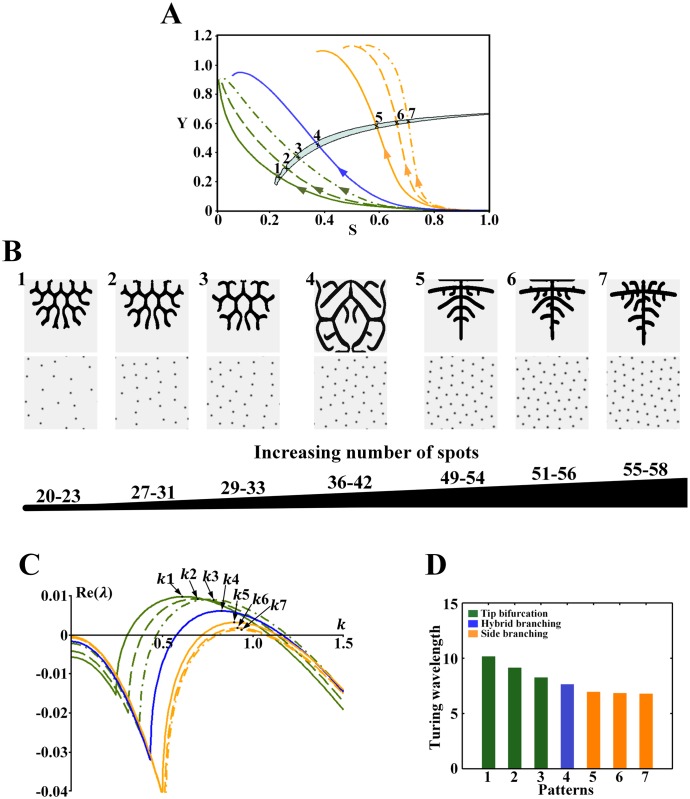
Effect of the wavelength on the branching patterns in the Turing region for *ρ*_*H*_ = 0.00007. (A) The cell differentiation trajectories of the branching patterns cross the Turing region. Points 1–7 correspond to the position of the underlying Turing patterns. (B) Branching patterns (top) and underlying spot patterns (bottom). Patterns 1–7 correspond to points 1–7 in A. (C) Dispersion relations for patterns, with wavenumber *k*1-6 corresponding to patterns 1–7 in B. (D) Comparison of the wavelength (2π/wavenumber) from patterns 1–7 in B. Parameters: *c* = 0.002, *μ* = 0.16, *ρ*_*A*_ = 0.03, *D*_*A*_ = 0.02, *v* = 0.04, *ρ*_*H*_ = 0.00007, *D*_*H*_ = 0.3, *c*_0_ = 0.02, *D*_*S*_ = 0.06, (B1-7) *ε* = 1.5/1.2/0.9/0.45/0.1/0.06/0.045. In the 2D patterns, black indicates a high concentration, and gray indicates a low concentration.

**Fig 10:**
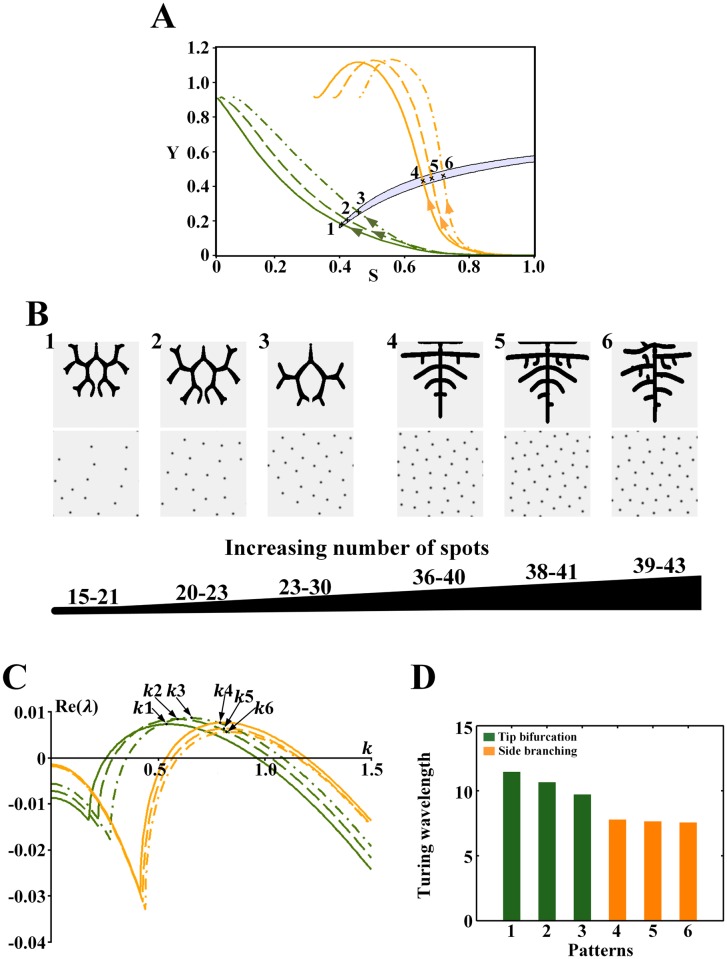
Effect of the wavelength on the branching patterns in the Turing region for *ρ*_*H*_ = 0.00013. (A) The cell differentiation trajectories of the branching patterns cross the Turing region. Points 1–6 correspond to the position of the underlying Turing patterns. (B) Branching patterns (top) and underlying spot patterns (bottom). Patterns 1–6 correspond to points 1–6 in A. (C) Dispersion relations for patterns, with wavenumber *k*1-6 corresponding to patterns 1–6 in B. (D) Comparison of the wavelength (1π/wavenumber) from patterns 1–6 in B. Parameters: *c* = 0.002, *μ* = 0.16, *ρ*_*A*_ = 0.03, *D*_*A*_ = 0.02, *v* = 0.04, *ρ*_*H*_ = 0.00013, *D*_*H*_ = 0.3, *c*_0_ = 0.02, *D*_*S*_ = 0.06, (B1-6) *ε* = 1.1/0.85/0.7/0.07/0.06/0.045. In the 2D patterns, black indicates a high concentration, and gray indicates a low concentration.

**Fig 11:**
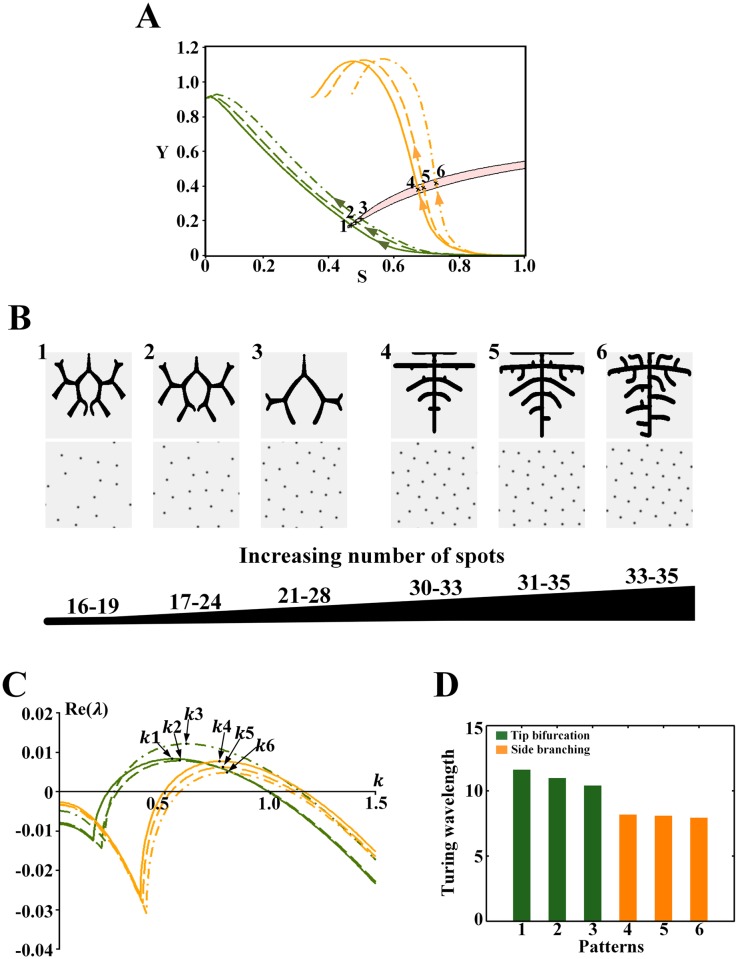
Effect of the wavelength on the branching patterns in the Turing region for *ρ*_*H*_ = 0.00015. (A) The cell differentiation trajectories of the branching patterns cross the Turing region. Points 1–6 correspond to the position of the underlying Turing patterns. (B) Branching patterns (top) and underlying spot patterns (bottom). Patterns 1–6 correspond to points 1–6 in A. (C) Dispersion relations for the patterns, with wavenumber *k*1-6 corresponding to patterns 1–6 in B. (D) Comparison of the wavelength (2π/wavenumber) from patterns 1–6 in B. Parameters: *c* = 0.002, *μ* = 0.16, *ρ*_*A*_ = 0.03, *D*_*A*_ = 0.02, *v* = 0.04, *ρ*_*H*_ = 0.00015, *D*_*H*_ = 0.3, *c*_0_ = 0.02, *D*_*S*_ = 0.06, (B1-6) *ε* = 0.9/0.8/0.65/0.07/0.06/0.045. In the 2D patterns, black indicates a high concentration, and gray indicates a low concentration.

To investigate the effect of the wavelength on the branching patterns for different Turing regions, we varied parameter *ρ*_*H*_ (in Eq (2), inhibitor secreted by cells) to explore the different Turing regions, since *ρ*_*H*_ is a key factor in the branching patterns in the simulation[5] and is a parameter in the activator-inhibitor model (Eqs (1) and (2)). As we have already analyzed the Turing region for *ρ*_*H*_ = 0.0001, four Turing regions for two smaller and two larger *ρ*_*H*_ values are selected for the analysis. Fig 7 shows five Turing regions for *ρ*_*H*_ = 0.00005, 0.0007, 0.0001, 0.00013, and 0.00015. For each Turing region, we also obtained simulation results of the branching patterns and the underlying spot patterns by varying *ε* and analyzed the wavelength through dispersion relations. The results for the four Turing regions are shown in Figs 8–11, respectively.

Fig 8 shows the effect of the wavelength on the branching patterns and the underlying spot patterns in the Turing region for *ρ*_*H*_ = 0.00005. Fig 8A shows the Turing region for *ρ*_*H*_ = 0.00005, and the branching patterns and their underlying spot patterns are shown in Fig 8B. Through dispersion relation analysis (Fig 8C), we show the wavelength (Fig 8D). Trends similar to those for the Turing region with *ρ*_*H*_ = 0.0001 were observed. For the tip bifurcation patterns, when the bifurcation rate decreases and the number of spots in the underlying sparse spot patterns increases (Fig 8B), the wavelength decreases (Fig 8D, green bars). When the branching mode switches from tip bifurcation to side branching with the underlying spot patterns changing from a sparse to a dense distribution (Fig 8B), the wavelength decreases further (Fig 8D, green to orange bars). For the side branching patterns, when the spatial interval between branches decreases and more branches grow outward, the number of spots in the underlying dense spot patterns increases slightly (Fig 8B), and the wavelength decreases slowly (Fig 8D, orange bars).

In addition to the similar trends, an interesting hybrid branching pattern (Fig 8B4), which mixes tip bifurcation and side branching, is generated. Both the number of spots and the wavelength of the underlying spot patterns are between those corresponding to the tip bifurcation and side branching patterns (Fig 8B4 and 8D, blue bars).

For the Turing region for *ρ*_*H*_ = 0.00007, similar trends are observed in Fig 9. For the tip bifurcation patterns, when the bifurcation rate decreases and the number of spots in the underlying sparse spot patterns increases (Fig 9B), the wavelength decreases (Fig 9D, green bars). When the branching mode switches to side branching with the underlying dense spot patterns (Fig 9B), the wavelength decreases further (Fig 9D, green to orange bars). For the side branching patterns, when the branches grow closely and the number of spots in the underlying dense spot patterns increases slightly (Fig 9B), the wavelength decreases slowly (Fig 9D, orange bars).

Similarly, a hybrid branching pattern also emerges, and both the number of spots and the wavelength of the underlying spot patterns are between those corresponding to the tip bifurcation and side branching patterns (Fig 9B4 and 9D, blue bars). However, Fig 9B4 shows that it is not easy for side branch to emerge from the tip branching structure.

Similar trends were found in the Turing region for *ρ*_*H*_ = 0.00013 in Fig 10. For the tip bifurcation patterns, when the bifurcation rate decreases and the number of spots in the underlying sparse spot patterns increases (Fig 10B), the wavelength decreases (Fig 10D, green bars). When the branching mode switches to side branching with a dense underlying spot pattern (Fig 10B), there is an evident decrease in the wavelength (Fig 10D, green to orange bars). For side branching patterns, when branches grow close and the number of spots in the underlying dense spot patterns increases slightly (Fig 10B), the wavelength decreases slowly (Fig 10D, orange bars).

However, no hybrid branching patterns were observed in the Turing region for 0.00013.

Similar trends were observed in Fig 11 in the Turing region for *ρ*_*H*_ = 0.00015. When the bifurcation rate in the tip bifurcation patterns decreases and the number of spots in the underlying sparse spot patterns increases (Fig 11B), the wavelength decreases (Fig 11D, green bars). When the branching mode switches to side branching with an underlying dense spot pattern (Fig 11B), the wavelength decreases greatly (Fig 11D, green to orange bars). In the side branching patterns, when branches grow close and the number of spots in the underlying dense spot patterns increases slightly (Fig 11B), the wavelength decreases slowly (Fig 11D, orange bars).

Additionally, there are no hybrid branching patterns observed in the Turing region for *ρ*_*H*_ = 0.00015.

The simulation results demonstrate that the effects of the wavelength on the branching patterns have similar trends in different Turing regions. For the tip bifurcation patterns, when the bifurcation rate decreases and the number of spots in the underlying sparse spot pattern increases, the wavelength decreases. When the branching mode switches from tip bifurcation to side branching and the underlying spot pattern changes from a sparse to dense distribution, the wavelength decreases further. For the side branching patterns, when the spatial interval between branches decreases and the number of spots in the underlying dense spot pattern increases slightly, the wavelength decreases slowly.

## Discussion

Our simulation results demonstrate that a local high morphogen concentration and the Turing wavelength play important roles in pattern formation in the branching model.

In the branching patterns, the growing tips exhibit Turing instability. We show that it is the Turing spot patterns underlying the branching patterns. The spot patterns are in the form of concentration peaks, which results in a local morphogen concentration peak formed at the tips in the branching patterns. The local morphogen concentration peak is unstable and induces tip expansion into the free space, causing branches to grow. This result is in agreement with the in vitro experimental results of Hagiwara *et al*[18], who showed that a high cell concentration gradient is required for cell branching in the lung.

Furthermore, we found that the spot density of the spot pattern varies for branching structures. A sparse spot pattern underlies the tip bifurcation patterns, while a dense spot pattern underlies the side branching patterns.

The dispersion relation analysis shows that the wavelength of the spot patterns affects the occurrence of tip bifurcation and side branching. For the tip bifurcation patterns, when the bifurcation rate decreases and the number of spots in the underlying sparse spot pattern increases, the wavelength decreases. When the branching mode switches from tip bifurcation to side branching and the underlying spot pattern changes from a sparse to a dense distribution, the wavelength decreases further. For the side branching patterns, when the spatial interval between branches decreases as more branches grow, the number of spots in the underlying dense spot pattern increases slightly and the wavelength decreases slowly.

The simulation results suggest that when the wavelength decreases and the number of spots in the spot patterns increases, tip bifurcation occurs at a decreasing rate. When the wavelength decreases below a certain value, the spot patterns shift to a dense distribution, no tip bifurcation occurs and side branching is observed. An insufficient wavelength impedes tip bifurcation but provides favorable conditions for side branching.

Branching patterns and the Turing patterns are two types of patterns in mathematical biology. Our work contributes to correlating the formation of branching patterns with Turing patterns. Although we demonstrate the connection between spot patterns and branching patterns, little is known about other Turing patterns, such as stripe patterns and hole patterns. The dispersion relation analysis shows that the wavelength affects the branching pattern, and the trend of how the wavelength affects the branching structures is revealed. However, the exact mechanism remains to be explored.

Nevertheless, our work reveals the Turing mechanism underlying the branching patterns. In our previous study[5], we demonstrated that the branching mode can be changed from tip bifurcation to side branching by varying the parameter *ε*. Our results in this paper further show that *ε* controls the branching mode switch because it regulates the Turing wavelength. In the experimental work, the branching mode changes during lung development are shown to be controlled by genes. Our results further suggest that the branching mode switch in the lung is a result of genes regulating the Turing wavelength, similar to a previous study [19], which found that gene modulation of digit patterning involves a Turing mechanism. Our work provides a fresh insight into and understanding of the formation of branching patterns in the lung and other biological branching systems.

## Appendix. Linear stability analysis of the activator-inhibitor model

To calculate the S-Y parameter space of the activator-inhibitor model for the Turing instability, linear stability analysis [7, 8, 20–22] is performed. The activator-inhibitor model is defined by

$$\{\begin{array}{c} \frac{\partial A}{\partial t}=\frac{cA^{2}S}{H}-\mu A+\rho_{A}Y+D_{A}\nabla^{2}A\\ \frac{\partial H}{\partial t}=cA^{2}S-vH+\rho_{H}Y+D_{H}\nabla^{2}H \end{array}$$ (A1)

Denote

$$f(A,H)=\frac{cA^{2}S}{H}-\mu A+\rho_{A}Y,\;g(A,H)=cA^{2}S-vH+\rho_{H}Y.$$ (A2)

We are interested in the positive equilibrium of System (A1). By solving the relation:

$$\{\begin{array}{c} f(A,H)=0\\ g(A,H)=0 \end{array}$$ (A3)

We can obtain the positive equilibrium denoted by $\;E^{*}=\left(\begin{array}{c} A^{*}\\ H^{*} \end{array}\right)$. Then, we make the following nonuniform perturbations from E*:

$$\left(\begin{array}{c} A\\ H \end{array}\right)=\left(\begin{array}{c} A^{*}\\ H^{*} \end{array}\right)+\zeta \left(\begin{array}{c} A_{k}\\ H_{k} \end{array}\right)e^{\lambda t+i\text{kr}}+c.c.+O(\zeta^{2}),$$ (A4)

where *λ* is the growth rate of the perturbations over time *t*, *i* is an imaginary unit, ***k*** is the wave vector, ***r*** is the spatial vector in two-dimensional space and c.c. represents the complex conjugate. By substituting Eq (A4) into System (A1), we obtain the characteristic equation:

$$\left(\begin{array}{cc} r_{11}-k^{2}D_{A}-\lambda & r_{12}\\ r_{21} & r_{22}-k^{2}D_{H}-\lambda \end{array}\right)=0,$$ (A5)

where

$$\begin{array}{l} r_{11}={\frac{\partial f}{\partial A}|}_{A^{*},H^{*}}=\frac{2cA^{*}S}{H^{*}}-\mu ,r_{12}={\frac{\partial f}{\partial H}|}_{A^{*},H^{*}}=-\frac{cA^{*2}S}{H^{*2}},\\ r_{21}={\frac{\partial g}{\partial A}|}_{A^{*},H^{*}}=2cA^{*}S,r_{22}={\frac{\partial g}{\partial H}|}_{A^{*},H^{*}}=-v. \end{array}$$

The characteristic equation is equivalent to the following equation:

$$\lambda^{2}-tr_{k}\lambda +\Delta_{k}=0,$$ (A6)

where

$$\begin{array}{l} tr_{k}=r_{11}+r_{22}-k^{2}(D_{A}+D_{H}),\\ \Delta_{k}=r_{11}r_{22}-r_{12}r_{21}-k^{2}(r_{11}D_{H}+r_{22}D_{A})+k^{4}D_{A}D_{H} \end{array}$$ (A7)

Eigenvalues *λ*_*k*_ are:

$$\lambda_{k}=\frac{tr_{k}\pm \sqrt{tr_{k}{}^{2}-4\Delta_{k}}}{2}$$ (A8)

According to Turing theory, Turing instability occurs in System (A1) if *Re*(*λ*_*k* = 0_) < 0 and *Re*(*λ*_*k*≠0_) > 0 for some *k*. These conditions require

$$\begin{array}{l} r_{11}+r_{22}<0,\\ r_{11}r_{22}-r_{12}r_{21}>0,\\ r_{11}D_{H}+r_{22}D_{A}>0,\\ {\left(r_{11}D_{H}+r_{22}D_{A}\right)}^{2}-4D_{A}D_{H}(r_{11}r_{22}-r_{12}r_{21})>0. \end{array}$$ (A9)

We take S and Y as the controlled parameters. Therefore, inequalities Eq (A9) define a domain in (S, Y) parameter space, namely, the Turing region. The expression *λ* = *λ*(*k*) is called a dispersion relation given by Eq (A8). Analysis of the dispersion relation is thus informative because it shows the features of the spatial patterns. We conduct an analysis of the dispersion relation for the Turing patterns underlying the branching patterns in the results section.

## Supporting information

S1 Code Numerical simulation of the branching patterns. Numerical simulations were written in CUDA for GPU implementation.
(CU) Click here for additional data file.

S2 Code Numerical simulation of the Turing patterns. Numerical simulations were written in CUDA for GPU implementation.
(CU) Click here for additional data file.
